# Current practices in the management of closed femoral shaft fractures in children: A nationwide survey among Dutch orthopaedic surgeons

**DOI:** 10.1016/j.jor.2023.09.008

**Published:** 2023-09-23

**Authors:** Stijn van Cruchten, Eefke C. Warmerdam, Max Reijman, Dagmar RJ. Kempink, Victor A. de Ridder

**Affiliations:** a UMC Utrecht, Heidelberglaan 100, 3584 CX, Utrecht, the Netherlands; b Reinier de Graaf Gasthuis, Reinier de Graafweg 5, 2625 AD, Delft, the Netherlands; c Erasmus MC Sophia Children Hospital, Wytemaweg 80, 3015 CN, Rotterdam, the Netherlands

**Keywords:** Femur shaft fractures, Pediatric, Intramedullary nails, Spica cast, Traction, Titanium elastic nails

## Abstract

**Background:**

There remains a lack of high-quality evidence on the treatment of pediatric femur shaft fractures. Therefore, treatment choices may still be based on personal preference of treating surgeons. To gain insight in considerations regarding treatment options, we conducted a survey among Dutch trauma and orthopedic surgeons.

**Methods:**

This survey was conducted in 2020, regarding treatment considerations for closed femoral shaft fractures in children in different age and weight groups.

**Results:**

One hundred forty-two surgeons were included in the analysis. 31% of participating surgeons considers surgical fixation in children of 2–4 years old, compared to 83% in children of 4–6 years old. In terms of weight, 30% considers surgery in children of 10–15 kg, compared to 77% considering surgery in children weighing 15–20 kg. While most surgeons find traction and spica cast suitable options for children younger than 4 years, a minority also considers these treatment modalities for children older than 4 (traction: 81% versus 19%, spica cast 63% versus 29% respectively). 33% of surgeons considers ESIN under 4 years of age, compared to 88% in children older than 4.

**Conclusion:**

An age of 4 years and a weight of 15 kg seem to be cut off points regarding preference of non-surgical versus surgical treatment of closed femoral shaft fractures. There is a wide range of ages and sizes for which treatment options are still being considered, sometimes differing from the national guideline. This questions guideline adherence, which may be due to a lack of available high-quality evidence.

## Highlights

• Almost all trauma and orthopedic surgeons take both age and size in consideration when deciding on treatment of pediatric closed femur shaft fractures.
• An age of 4 and a weight of 15 kg seem to be cut off points regarding preference of non-surgical versus surgical treatment of closed femoral shaft fractures.
• Although the identified turning points of 4 years old and 15 kg are in accordance with the national guideline, the wide range of ages and sizes for which some treatment options are still being considered might imply a limited adherence to this guideline.

## 1 Introduction

Several treatment options are available for the management of femoral shaft fractures in children. Still, these fractures pose a challenge to trauma and orthopedic surgeons: although not frequently encountered,^1^^,^^2^ they lead to significant disability^3^ and hospital admission.^4^ There is consensus that treatment should differ according to size and age. Younger children tend to be treated conservatively, while older children are more prone to surgical intervention.^5^ However, opinions differ on the exact age limit for non-operative treatment. A recent systematic review showed limited evidence for treatment of these fractures in the age group of 2–10 years old, although the results slightly favored treatment with elastic intramedullary nails (ESIN), even for the younger patients.^6^ The current Dutch trauma guideline on pediatric femoral shaft fractures also acknowledge this lack of evidence regarding treatment in the age group from 2 to 10 years old, the advice being to treat the youngest age groups non-operatively and to provide surgical treatment in children older than 4 years old.^7^ Still, this cut off point is not based on sufficient scientific evidence, and treatment decisions continue to be based on experience. Therefore, more insight in current practice is necessary. Are treatment choices based on age, size or both? Which treatment modalities are considered for different patient groups? Which treatment do our Dutch (trauma/orthopedic) surgeons currently choose for these children? For this cause, a survey was conducted among Dutch trauma surgeons and orthopedic surgeons. This article provides an overview of current management of femoral shaft fractures in children aged 0–10 years in the Netherlands.

## 2 Materials and methods

### 2.1 Survey methods

The survey was designed by one of the authors and independently peer reviewed by the others. The final version consisted of 33 questions and was divided into an introduction (4 questions), a section regarding suspicion of non-accidental injury (3 questions) and the main section covering various treatment modalities for femoral shaft fractures in children (26 questions) (see appendix). The introduction section asked the participating surgeons about their current hospital, their surgical experience and the incidence of pediatric femoral shaft fractures in their practice. We included section on non-accidental injury on surgeons’ reporting frequency of femoral shaft fractures suspicious of non-accidental injury. The survey questions in the main sections focused on preferences regarding the use of different treatment modalities in various age and weight groups. By consulting current guidelines and published literature, common treatment modalities were found to be Pavlik bandage, traction, traction and subsequent spica casting, immediate spica casting, ESIN, plate fixation, external fixation and intramedullary locking nail. These were included in the survey questions of the main section. For every treatment modality respondents were asked whether they considered a treatment option and for which specific age categories and weight categories. The complete survey is displayed in Addendum 1.

A pilot survey was executed among trauma and orthopedic surgeons (n = 5) to evaluate logic and length of the survey. This pilot led to a small rearrangement of survey questions. The survey was found to have a suitable length with a mean completion time of 7 min.

The survey was generated in the electronic survey tool SurveyMonkey. Invitations were sent via two different response collectors: The author's received a database from the Dutch Traumatology Association (NVT) containing all known trauma surgeons regularly treating pediatric femur shaft fractures. On April 24, 2020, these surgeons were sent an email invitation to participate in the survey as well. Secondly, on June 5, 2020 a web link to the survey was sent via the NVOT (Dutch Association of Orthopedic Trauma) to orthopedic surgeons in the Netherlands. All responses were combined into one database, and this database was manually screened for duplicates.

Respondents were included in the study when they completed the whole questionnaire and treated femoral shaft fractures in the Netherlands in the 5 years prior to the questionnaire. Respondents were excluded when they did not complete the survey, or when they were not working as a surgeon in the Netherlands at the moment of participation.

For each question, we calculated percentages and presented descriptive statistics based on the combined responses of participating surgeons.

## 3 Results

In total, 154 surgeons and surgical residents responded to the survey. Surgeons were asked to participate through 1. Email invitation and 2. A web link invitation by SurveyMonkey. The email invitation was sent to 80 email addresses and led to 48 responses, thus yielding a response rate of 60.0%. The web link was sent to 572 surgeons and led to 106 responses, yielding a response rate of 18.5%. Overall, response rate was 23.6%. No duplicates were identified after manually screening all respondents.

Four respondents were excluded due to the following reasons: retirement (n = 1), employment by a hospital abroad (n = 1) and not completing the survey (n = 2). This left 150 respondents from 68 different hospitals or clinics. Table 1 shows the distribution of surgical experience among respondents. Eight respondents (6.6%) did not treat pediatric femur fractures in the past 5 years, which led to 142 respondents included in the analysis.

**Table 1:** Distribution of surgical experience of responding surgeons.

Surgical experience	No. of respondents	Percentage
Surgical resident	*5*	*3.52%*
0–5 years	*33*	*23.24%*
5–10 years	*23*	*16.90%*
>10 years	*81*	*57.04%*
Total	*142*	*100%*

**Fig. 1:**
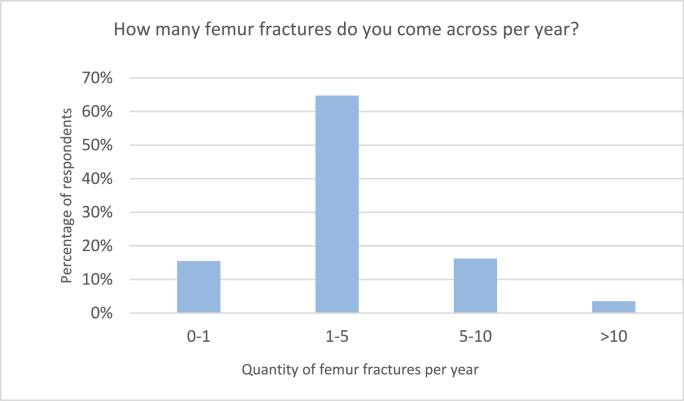
Responses to the survey question: “How many femur fractures do you come across per year?”

Of 142 respondents, twenty-two (15.5%) treated a mean 0–1 pediatric femur fractures per year, ninety-two respondents (64.8%) report a mean of 1–5 fractures, twenty-three respondents (16.2%) saw 5–10 fractures per year and five (3.5%) reported treatment of 10+ pediatric femur fractures per year (Figure 1).

### 3.1 Treatment considerations

Although most respondents treat these fractures themselves (93.7%), six respondents (4.2%) refer these children to other hospitals. Three respondents (2.1%) declared they start treatment, but subsequently refer for further treatment. When deciding on treatment 95% of the participating surgeons take a patient's age into consideration, and 86% a patient's weight.

### 3.2 Pavlik

Thirty percent of Dutch surgeons considers Pavlik bandage for treating femur fractures. Only 21% of these surgeons considers Pavlik bandage for children older than 6 months old. And only 19% for children weighing more than 5–10 kg.

### 3.3 Traction

**Fig. 2:**
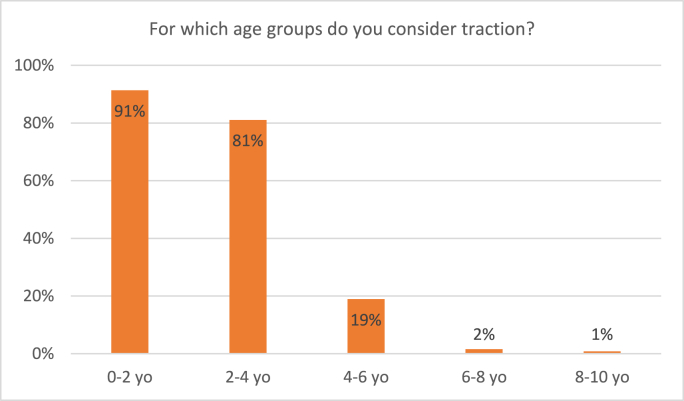
Responses to the survey question: “For which age groups do you consider traction?” Respondents were able to tick multiple boxes.

**Fig. 3:**
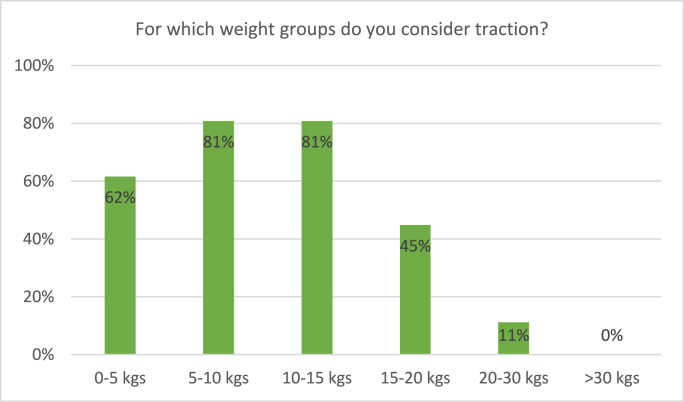
Responses to the survey question: “For which weight groups do you consider traction?” Respondents were able to tick multiple boxes.

Ninety-five percent of participating surgeons considers traction for pediatric femur shaft fractures. Figure 2 shows that in the age groups of 2–4 years old, 81% of respondents see traction as a treatment option. For children of 4–6 years old, only 19% considers traction. For weight, 81% considers traction for children weighing between 5 and 15 kgs. In children heavier than 15 kgs, a minority of participating surgeons would consider traction (figure 3).

### 3.4 Spica casting

**Fig. 4:**
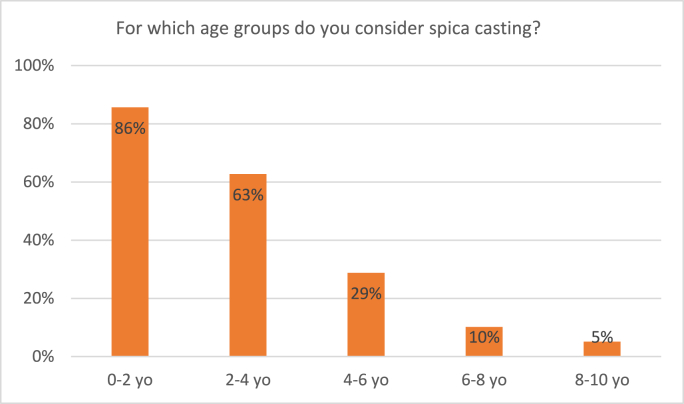
Responses to the survey question: “For which age groups do you consider spica casting?” Respondents were able to tick multiple boxes.

**Fig. 5:**
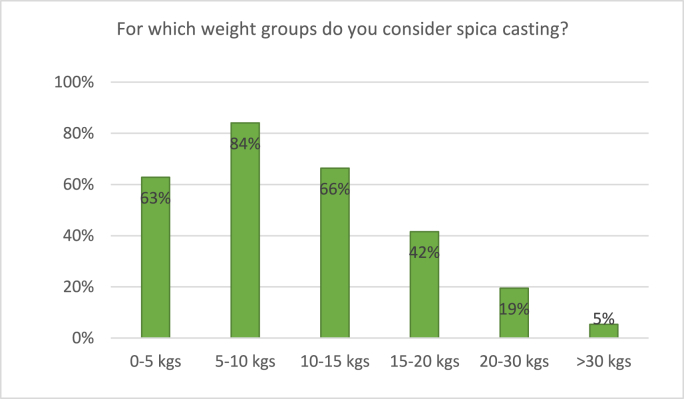
Responses to the survey question: “For which weight groups do you consider spica casting?” Respondents were able to tick multiple boxes.

Ninety percent of participating surgeons considers spica casting for pediatric femur shaft fractures. 63% considers spica casting for children aged 2–4 years compared to 29% in children aged 4–6 years (figure 4). In terms of weight, considerations of participating surgeons are depicted in figure 5. A majority of surgeons considers spica casting in children weighing up to 15 kgs, compared to 42% of surgeons in children of 15–20 kgs.

### 3.5 Surgical fixation

**Fig. 6:**
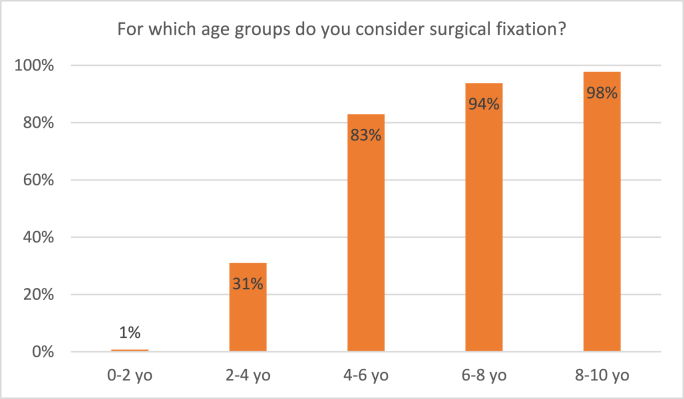
Responses to the survey question: “For which age groups do you consider surgical fixation?” Respondents were able to tick multiple boxes.

**Fig. 7:**
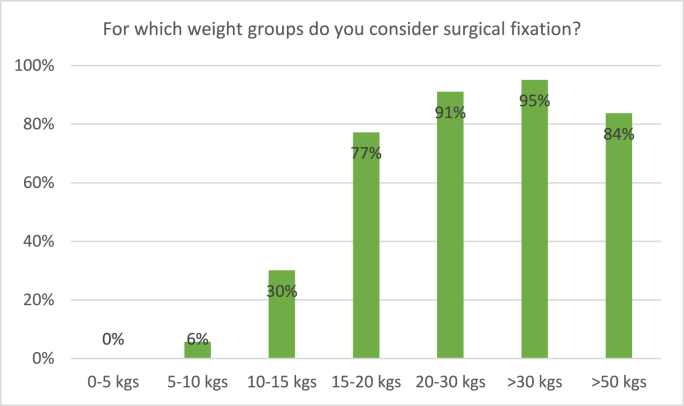
Responses to the survey question: “For which weight groups do you consider surgical fixation?” Respondents were able to tick multiple boxes.

The majority of surgeons declared surgical treatment a suitable treatment option in children from the age of 4: 31% considers surgery in 2-4-yearolds, 83% in 4-6-yearolds (figure 6). Figure 7 depicts the respondents’ considerations regarding weight: In children weighing 10–15 kgs, only 30% of surgeons considers surgery, compared to 77% in children of 15–20 kgs.

### 3.6 Elastic intramedullary nails

**Fig. 8:**
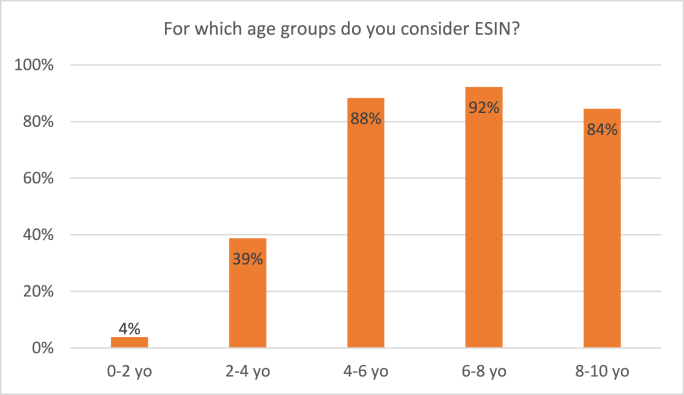
Responses to the survey question: “For which age groups do you consider ESIN?” Respondents were able to tick multiple boxes.

**Fig. 9:**
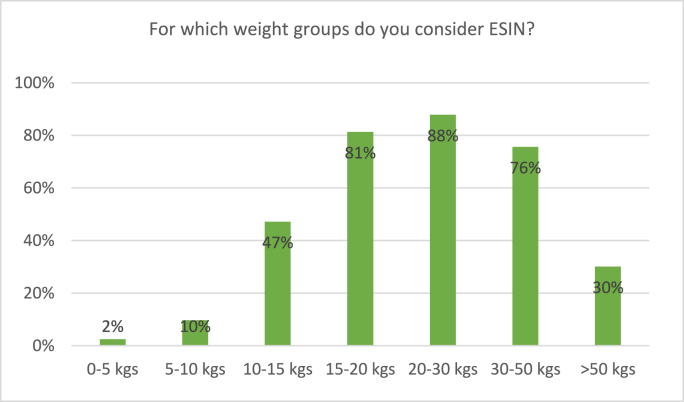
Responses to the survey question: “For which weight groups do you consider ESIN?” Respondents were able to tick multiple boxes.

All participating surgeons consider treatment with ESIN for pediatric femoral shaft fractures. Figure 8 depicts survey results regarding treatment with ESIN. Thirty-nine percent of respondents would consider treatment with ESIN in children of 2–4 years old compared to 88% in children of 4–6 years old. In children weighing 10–15 kgs, 47% of surgeons would consider ESIN for treatment, as compared to 81% in 15–20 kgs. Still 30% of surgeons consider ESIN for children weighing heavier than 50 kgs (Fig. 9).p.

### 3.7 Plate fixation

Sixty-seven percent of participating surgeons considers plate fixation for pediatric femur shaft fractures. Twenty-six percent considers it in children of 4–6 years, 62% in children of 6–8 and 100% thought of it as a possible option for children older than 10 years old. The rate of surgeons considering plate fixation increased as patient weight increases: 52% in 20–30 kgs, and 80% in 30–50 kg.

### 3.8 Intramedullary locking nail

Only 24% of surgeons considers intramedullary locking nail. Of these surgeons, 16% do so in children of 6–8 years old, and 100% in the age group of 8–10 years. In children weighing 30–50 kgs, 36% considers a locking nail, compared to 64% in children of more than 50 kgs.

### 3.9 External fixator

External fixation is considered only by a minority of surgeons: 27.5% considers external fixation in case of an open fracture, and 45% considers it in case of polytrauma. 27.5% percent declared they would never use an external fixator in children with femoral shaft fractures.

## 4 Discussion

Although most guidelines provide a general recommendation on treatment of pediatric femoral fractures, authors acknowledge that no clear consensus has been reached regarding optimal treatment.^7^^,^^8^ This is the first survey in recent years on how femoral shaft fractures in children aged 2–10 years are currently managed in a high-income country.

The Dutch national pediatric fracture guideline by the Dutch Federation of Medical Specialists recommends treatment with traction, possibly followed by spica casting in children of 3 months–4 years of age, and ESIN in children of 4–12 years of age weighing no more than 50 kgs.^7^

Generally, our main findings are in accordance with this guideline. There was a clear trend toward surgical fixation from the age of 4 onward: only 31% considered surgical fixation in children of 2–4 years old, compared to 83% in children of 4–6 years old. For weight, a similar trend was noticeable between 10 and 15 kgs (30%) and 15–20 kgs (77%).

Nearly all respondents declared that they treat these fractures themselves, instead of referring to other hospitals. Almost all of them consider traction, spica casting and ESIN for treatment.

For these treatment modalities, the age of four was found to be a cutoff point; while the majority of surgeons finds traction or spica casting suitable treatment options for children under 4 years of age, only a minority still considers these in children over the age of 4 (traction: 81% vs 19%; spica 63% vs 29%). For ESIN, this is inversed, with a minority (33%) considering ESIN under 4 years old and 88% in children older than 4. This is in line with a previous survey of Curran et al. whom investigated treatment patterns of pediatric femur fractures in low- and middle-income countries. In high-income countries, as are the Netherlands, there was a clear tendency to conservative treatment in children up to 4 years of age: Respondents of 79% of these countries chose for non-operative treatment in the majority of patients. In children of 5–12 years of age, the majority of respondents of 76% of these countries chose surgical treatment, of which approximately 79% with intramedullary nails. What seems to be in contrast with our findings, is that 80% of conservative treatment under 4 years of age consisted of spica casting, and only 20% of either traction or traction with subsequent casting.^9^ In our survey, the rates of surgeons considering traction were higher than for spica casting. In 1998, Sanders et al. conducted a survey among orthopedic surgeons in several high-income countries. Because treatment patterns have changed significantly in the last 20 years, patients were divided into different age groups, and the results were further specified for different fracture patterns, the results may not be directly applicable to our study. Still, they also found an increasing preference for operative treatment with age.^10^

Regarding weight, non-operative treatment with either traction or spica casting was considered by a majority of surgeons in children weighing up to 15 kgs of weight, which seemed to be a cutoff point. In children between 10 and 15 kgs, traction was considered by 81% and spica cast by 66%, compared to respectively 45% and 42% in children of 15–20 kgs. Only a small part of surgeons would still consider non-operative treatment in children weighing over 20 kgs. This is in accordance with the answers regarding surgical fixation: only 30% of surgeons would consider surgical fixation in children weighing 10–15 kgs, compared to 77% in the 15–20 kgs group, and even 91% in the 20–25 kgs group. ESIN specifically would be considered by a majority of surgeons for children weighing in between 15 and 50 kgs. We found no previous surveys on weight as a treatment consideration for pediatric femur fractures. It should be noted that findings concerning age- and weight-depending treatment choices are merely applicable for Dutch children and children in countries of comparable growth and weight patterns. We published a meta-analysis on this subject in 2021, presenting all available evidence on treatment of pediatric femur shaft fractures in children aged 2–10 years. This meta-analysis included a subgroup analysis of children of 2–6 years old, and showed a tendency to elastic intramedullary nailing for both this subgroup as the whole patient group. Unfortunately, we were unable to further specify age groups, and it remains unclear whether treatment with intramedullary nails would be preferable for children under 4 years old. Still, these conclusions are in accordance with this survey's results, as both studies found that surgical treatment is preferred over conservative treatment as from relatively young age.^6^

A noticeable finding is the wide range in ages and sizes that certain treatment modalities are being considered for. For instance, 29% of surgeons considers treatment with spica cast in children of 4–6 years old, 39% of surgeons considers surgical fixation with ESIN of femur shaft fractures in children of 2–4 years of age and 30% considers ESIN fixation in children weighing over 50 kg. These findings are not in accordance with the national guideline. These outliers could be explained by the consideration of treatment options for patients that differ from the mean size at certain ages. For example, heavy weighing children might be treated surgically at a younger age. Still, these findings may be partially explained by a lack of adherence to the national guideline.

This study has several strengths and weaknesses. Compared to previous literature, this is the only recent survey-based study on treatment of pediatric femur fractures in a high-income country. Also, it is the first to include weight as a treatment consideration. Furthermore, the survey was directed to both trauma surgeons and orthopedic surgeons, to prevent possible selection bias.

Possible limitations of this study are largely attributed to the fixed survey design, consisting mainly of “check all that apply” survey questions. Possible nuances in the respondents’ answers may have been filtered out. Also, because trauma surgeons were asked to participate by means of an email invitation, we were not able to contact all trauma surgeons in the Netherlands. This may have induced bias. Finally, our survey did not distinguish between fracture types and patterns.

## 5 Conclusion

Although several guidelines provide recommendations on treatment of pediatric femur shaft fractures in children, there remains a lack of high-quality evidence. Treatment decisions may not only be based on evidence, but experience as well. This survey presents treatment considerations of 142 trauma and orthopedic surgeons in the Netherlands. Almost all respondents take both age and weight into consideration when deciding on treatment. The age of 4 and a weight of 15 kgs. seem to be cutoff points in preference from conservative to surgical treatment. The majority of surgeons considers treatment with ESIN in children weighing between 15 and 50 kgs. In general, these results are in accordance with national guideline recommendations. However, there is a wide variety in age and size for which certain treatment modalities are still being considered. Some of these findings differ from what is recommended in the national guideline. This questions guideline adherence, which may be due to a lack of high-quality evidence. Therefore, further research is required to make management of pediatric femur shaft fractures more evidence based.

## Funding/sponsorship

This research did not receive any specific grant from funding agencies in the public, commercial or not-for-profit sectors.

## Informed consent

Not applicable.

## Institutional Ethical Committee approval

As this was merely a survey-based study among surgeons and no patients were involved in this study, no official approval was obtained bij the Institutional Ethical Committee.

## Authors contribution by use of CRediT roles*

Stijn van Cruchten Data curation, formal analysis, methodology, vizualisation, writing (original) Eefke Warmerdam Data Curation, Formal analysis, methodology, investigation, writing (review) Max Reijman Formal analysis, investigation, writing (review), validation Dagmar Kempink Investigation, conceptualization, writing (review) Victor de Ridder Conceptualization, methodology, supervision, writing (review).

* https://www.elsevier.com/authors/journal-authors/policies-and-ethics/credit-author-statement.

## Conflict of interest

None.
