# Supplementary material for: Current practices in the management of closed femoral shaft fractures in children: A nationwide survey among Dutch orthopaedic surgeons
Source: J Orthop. 2023 Sep 23;45:1–5. doi: 10.1016/j.jor.2023.09.008 (PMC10534205; doi:10.1016/j.jor.2023.09.008)
Supplement: Multimedia component 1 [file mmc1.docx]

Addendum 1: Survey

Introduction

1. Which hospital do you work at?
2. How long have you been a surgeon?
3. *I’m a resident*
4. *0-5 years*
5. *5-10 years*
6. *>10 years*
7. As a doctor, in the past 5 years have you seen femoral shaft fractures in children of 0 to 10 years old?
8. *Yes*
9. *No*
10. (following 3) How many have you seen on average per year?
11. *0-1*
12. *1-5*
13. *5-10*
14. *>10*

Child abuse

1. Have you considered reporting femoral shaft fractures in children under 2 years of age as possible child abuse?
2. *Yes*
3. *No*
4. (following 5) Have you ever actually reported child abuse in these children?

*a. Yes*

*b. No*

1. If you have ever reported child abuse in these children, how many did you report?

*In total: <open>*

*In the last 5 years: <open>*

Treatment

1. Do you treat pediatric femoral shaft fractures in your hospital?
   1. *Yes*
   2. *No*
2. Is the treatment of choice depending on the patient’s age?
   1. *Yes*
   2. *No*
3. Is the treatment of choice depending on the patient’s weight?
   1. *Yes*
   2. *No*
4. Do you consider treatment with traction for pediatric femur shaft fractures?
   1. Yes
   2. No
5. (following 11) If you do, for which age categories do you consider traction? You may select multiple answers.
   1. *0-2 years old*
   2. *2-4 years old*
   3. *4-6 years old*
   4. *6-8 years old*
   5. *8-10 years old*
6. (following 12) If you do, for which weight categories do you consider traction? You may select multiple answers.
   1. *0-5 kilograms*
   2. *5-10 kilograms*
   3. *10-15 kilograms*
   4. *15-20 kilograms*
   5. *20-30 kilograms*
   6. *>30 kilograms*
   7. *>50 kilograms*
7. Do you consider treatment with Pavlik harness for pediatric femur shaft fractures?
   1. *Yes*
   2. *No*
8. (following 14) If you do, for which age categories do you consider a Pavlik harness? You may select multiple answers.
   1. *0-6 months old*
   2. *6-12 months old*
   3. *1-2 years old*
9. (following 15) If you do, for which weight categories do you consider traction? You may select multiple answers.
   1. *0-5 kilograms*
   2. *5-10 kilograms*
   3. *10-15 kilograms*
10. Do you consider treatment with a spica cast for pediatric femor shaft fractures?
    1. *Yes*
    2. *No*
11. (following 17) If you do, for which age categories do you consider a spica cast? You may select multiple answers.
    1. *0-2 years old*
    2. *2-4 years old*
    3. *4-6 years old*
    4. *6-8 years old*
    5. *8-10 years old*
12. (following 18) If you do, for which weight categories do you consider a spica cast? You may select multiple answers.
    1. *0-5 kilograms*
    2. *5-10 kilograms*
    3. *10-15 kilograms*
    4. *15-20 kilograms*
    5. *20-30 kilograms*
    6. *>30 kilograms*
    7. *>50 kilograms*
13. If you consider treatment with a spica cast, at what point in treatment do you apply this cast? You may select multiple answers.
    1. *Immediately*
    2. *After 0-72 hours of traction*
    3. *After 72 hours to 1 week of traction*
    4. *After 1-2 weeks of traction*
    5. *After 2-4 weeks of traction*
    6. *After a surgical procedure*
    7. *Other answer: <open>*
14. Do you consider surgical fixation of pediatric femoral shaft fractures?
    1. *Yes*
    2. *No*
15. (following 21) If you do, for which age categories do you consider a spica cast? You may select multiple answers.
    1. *0-2 years old*
    2. *2-4 years old*
    3. *4-6 years old*
    4. *6-8 years old*
    5. *8-10 years old*
16. (following 22) If you do, for which weight categories do you consider surgical fixation? You may select multiple answers.
    1. *0-5 kilograms*
    2. *5-10 kilograms*
    3. *10-15 kilograms*
    4. *15-20 kilograms*
    5. *20-30 kilograms*
    6. *>30 kilograms*
    7. *>50 kilograms*
17. Do you consider treatment with external fixation?
    1. *Yes, in open fractures*
    2. *Yes, in case of polytrauma*
    3. *Never*
18. Do you consider treatment with intramedullary nails like TEN (titanium elastic nails) for pediatric femoral shaft fractures?
    1. *Yes*
    2. *No*
19. (following 25) If you do, for which age categories do you consider elastic intramedullary nails? You may select multiple answers.
    1. *0-2 years old*
    2. *2-4 years old*
    3. *4-6 years old*
    4. *6-8 years old*
    5. *8-10 years old*
20. (following 26) If you do, for which weight categories do you consider elastic intramedullary nails? You may select multiple answers.
    1. *0-5 kilograms*
    2. *5-10 kilograms*
    3. *10-15 kilograms*
    4. *15-20 kilograms*
    5. *20-30 kilograms*
    6. *>30 kilograms*
    7. *>50 kilograms*
21. Do you consider treatment with plate fixation for pediatric femur shaft fractures?
    1. *Yes*
    2. *No*
22. (following 28) If you do, for which age categories do you consider plate fixation? You may select multiple answers.
    1. *0-2 years old*
    2. *2-4 years old*
    3. *4-6 years old*
    4. *6-8 years old*
    5. *8-10 years old*
23. (following 29) If you do, for which weight categories do you consider plate fixation? You may select multiple answers.
    1. *0-5 kilograms*
    2. *5-10 kilograms*
    3. *10-15 kilograms*
    4. *15-20 kilograms*
    5. *20-30 kilograms*
    6. *>30 kilograms*
    7. *>50 kilograms*
24. Do you consider treatment with lateral entry pin for femoral shaft fractures?
    1. *Yes*
    2. *No*
25. (following 31) If you do, for which age categories do you consider a lateral entry pin? You may select multiple answers.
    1. *0-2 years old*
    2. *2-4 years old*
    3. *4-6 years old*
    4. *6-8 years old*
    5. *8-10 years old*
26. (following 32) If you do, for which weight categories do you consider a lateral entry pin? You may select multiple answers.
    1. *0-5 kilograms*
    2. *5-10 kilograms*
    3. *10-15 kilograms*
    4. *15-20 kilograms*
    5. *20-30 kilograms*
    6. *>30 kilograms*
    7. *>50 kilograms*
